# Network flow and flood routing model for water resources optimization

**DOI:** 10.1038/s41598-022-06075-0

**Published:** 2022-03-10

**Authors:** Ayoub Tahiri, Daniel Che, David Ladeveze, Pascale Chiron, Bernard Archimède

**Affiliations:** 1 Université de Toulouse, INP-ENIT, Laboratoire Génie de Production, LGP, Tarbes, France; 2 Compagnie d’Aménagement des Coteaux de Gascogne, Tarbes, France; 3 Department of Civil Engineering, Ohio University, Ohio, USA

**Keywords:** Hydrology, Scientific data, Software, Complex networks, Fluid dynamics, Design, synthesis and processing

## Abstract

Real-time management of hydraulic systems composed of multi-reservoir involves conflicting objectives. Its representation requires complex variables to consider all the systems dynamics. Interfacing simulation model with optimization algorithm permits to integrate flow routing into reservoir operation decisions and consists in solving separately hydraulic and operational constraints, but it requires that the water resource management model is based on an evolutionary algorithm. Considering channel routing in optimization algorithm can be done using conceptual models such as the Muskingum model. However, the structure of algorithms based on a network flow approach, inhibits the integration of the Muskingum model in the approach formulation. In this work, a flood routing model, corresponding to a singular form of the Muskingum model, constructed as a network flow is proposed and integrated into the water management optimization. A genetic algorithm is involved for the calibration of the model. The proposed flood routing model was applied on the standard Wilson test and on a 40 km reach of the Arrats river (southwest of France). The results were compared with the results of the Muskingum model. Finally, operational results for a water resource management system including this model are illustrated on a rainfall event.

## Introduction

One of the most important aspects of minimizing the impacts of flooding is the proper operation of flood control systems itself^1^. The hydraulic system includes rivers and their tributaries, catchments, and natural or artificial hydraulic structures. Operation management of these hydraulic system is very challenging since it involves conflicting objectives and complicated variables. Real-time management consists in maximizing benefits, minimizing costs, satisfying the required flows in the river and storing water in reservoirs, answering to water demands, avoiding floods, and preserving the quality of water. These management requirements cause a need for a river basin optimization model that provides appropriate results.

Modeling choices for monitoring hydraulic system depend on numerous criteria linked to the studied case. Therefore, compromises have to be done among decision scale, precision needs, expected robustness, computing cost and so on. In this paper we focus on the case of hydraulic constraints for large scale and strongly influenced water resource systems. A flood routing approach that can be integrated in the mathematical formulation of an optimization model is proposed. The flood routing model was design in order to limit the number of iterated simulations and the complexity of the optimization problem to solve.

In this work, a comparison between this model and the Muskingum’s model for the Wilson standard test and for a real case study is provided. Some applications of this approach to several French rivers are also presented.

## State of the art

Real-time operation of multireservoir systems involves various operational, hydrologic, and hydraulic considerations^2^. For efficient operation, real-time management model should contain a flow routing procedure to predict the impacts of the observed and/or predicted inflows hydrographs on the downstream parts of the river system^3^. As flood waves travel from upstream to downstream, they attenuate and get delayed. Flood waves are subject to two principal movements: uniformly progressive flow and reservoir action. A uniformly progressive flow designates a shifting of the wave from upstream to downstream without a change in shape, which would occur only under ideal conditions. Reservoir action designates the modification of a flood wave by reservoir pondage. Flood routing is a technique that determines the flood hydrograph at a section of a river using an upstream hydrograph.

The optimization problem for the operation of multireservoir systems under flooding conditions can be stated as follows:

1 $$\begin{aligned} Minimize: z=f(h,Q) \end{aligned}$$

subject to

2 $$\begin{aligned} \begin{aligned}{}&Hydraulic\ constraints: g(h,Q)=0 \\&Bounds\ on\ discharges: {\underline{Q}} \le Q \le {\overline{Q}} \\&Bounds\ on\ elevations: {\underline{h}} \le h \le {\overline{h}} \\ \end{aligned} \end{aligned}$$

where *Q* is the flow rate, and *h* is the water surface elevation. Bars above and below a variable denote the upper and lower bounds for that variable, respectively. *g* is a function that describes the flow in the different components of the hydraulic system. The objective function *z* is to be minimized ans depends on the total flood damage. Different optimization methods were proposed in the litterature for the optimization of complex water resource systems. An optimization method suited to the case to be addressed, i. e. to solve Eq. (1), depends on the nature of the objective function and the constraints. Labadie^4^ and Yeh^5^ reviewed the state of the art in optimization of multireservoir systems management and operations .

The constraints of the model (Eq. 2) can be divided into two groups: the hydraulic constraints and the operational constraints. The consideration of hydraulic constraints is crucial for the quality of the operations, since release decisions are made upstream and the target areas are usually downstream. Hence, every water resource optimization model should take into consideration the attenuation of the wave during the transfer^6^.

Many models exist to represent the flood routing in a reach. Basic routing approaches may be classified into two main families: Hydraulic and conceptual approaches.

The hydraulic approach applies the governing Barre de Saint Venant equations represented by the continuity equation and the momentum equation, respectively^7^. The integration of the Barre de Saint Venant equations in the formulation of water optimization algorithms presents several difficulties since these partial differential equations are nonlinear, and their numerical resolution requires a large amount of calculation^8^. In order to integrate flow routing into the reservoir operation model, many researchers interfaced a simulation model with the optimization algorithm^2,9–12^ discussed the combination of simulation and optimization for real time flood operation for reservoir system and suggested the coupling of nonlinear optimization and simulation to close the gap between theory and practice. Wurbs^3^ discussed simulation, optimization and combined simulation-optimization modeling approaches and presented the strengths and weakness of the reviewed models.

**Figure 1:**
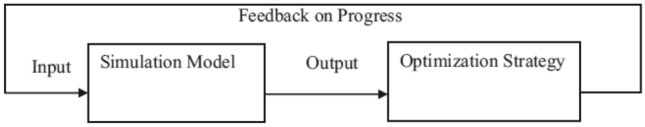
Interfacing a simulation model and an optimization algorithm.

The methodology of interfacing a simulation model to an optimization algorithm consists in solving separately the hydraulic and operational constraints: the optimization algorithm generates the optimal reservoir operating decisions, and the simulation model appropriately simulates the propagation of the flow for a given flood hydrograph and a set of operating decisions. Figure 1 presents the process of interfacing a simulation model and an optimization algorithm^13^. Simulation models produce outputs that are used by the optimization strategy to find an optimal solution.

Interfacing a simulation model with an optimization algorithm is only possible when the algorithm is based on an evolutionary computation approach^14^. The typical structure of evolutionary computation makes them suitable to adapt vaguely defined objective functions and constraints^15^. However, optimization-simulation process is time consuming and convergence problems may occur^16^. It is also to be noted that simulation models require: geometric data, initial condition, boundary condition, and hydraulic parameters which are not always available and bathymetric survey campaigns are very expensive. In addition, modeling biases can be observed that question the parameters of the model, and more so in the context of climate change^17,18^.

In the literature, the consideration of channel routing in optimization problem for the operation of multireservoir systems was also performed using conceptual routing procedures. Conceptual models are characterized by the fact that one does not seek to understand in detail the physical phenomena that occurs within the flow, but consider the network in its entirety; in other words, as a simple input-output transformer. The calibration of the model using input and output values allows fixing the parameters of the model. These models reflect only the consequences of the phenomena occurring in the system and therefore get over the difficulties of the hydraulic complexity. Most conceptual models are reservoir models; that is, the functioning of each reach is assimilated to the operation of one or more reservoirs in series or in parallel. Conceptual models are based on the continuity equation (the variation of the reach storage (*S*) corresponds to the difference between the inflow (*I*) and the outflow (*O*), see Eq. 3); and a second empirical relation (storage function, see Eq. 4) that connects the reach storage and the outflow rate^19^.

3 $$\begin{aligned} \frac{dS}{dt}=I-O \end{aligned}$$

The storage function can take many forms. Storage can be expressed as a function of inflow, outflow or both:

4 $$\begin{aligned} S=f\left( I,\frac{dI}{dt},\cdots ,O,\frac{dO}{dt},\cdots \right) \end{aligned}$$

The Muskingum flood routing model is the most used model^19^. The absolute storage is a function of both outflow and inflow discharges. The evolution in time of the absolute storage is expressed by:

5 $$\begin{aligned} S_t=K(xI_t+(1-x) O_t) \end{aligned}$$

where $S_t$ is the absolute reach storage at time $t; I_t$ and $O_t$ are the rates of inflow and outflow at time *t*, respectively; *K* is a coefficient with time dimension that represents the storage time of the reach or the travel time of flood waves through the channel reach; and *x* is a weighting dimensionless coefficient (between 0 and 0.5) that modulates the influence of the inflow and the outflow. Expressing Eqs. (3) and (5) in finite difference form for a time interval, while considering a transit time (*TT*) as additional pure delay, leads to:

6 $$\begin{aligned} O_t=C_0 I_{t-TT}+C_1I_{t-TT-1}+C_2O_{t-1} \end{aligned}$$

where $C_0$, $C_1$ and $C_2$ are constants that are computed from *K* and *x* and $\Delta T$^20^.

Muskingum flood routing model is widely used in optimization problem for the operation of multireservoir systems to represent channel routing, mainly because its formulation is linear and does not increase the complexity of the problem. Muskingum model has always been the first choice to model channel routing when the optimization problem is in a linear form. Windsor^21^ formulated a theoretical recursive linear programming model for the operation of flood control, using the Muskingum method for channel routing. Hsu and Wei^22^ developed a reservoir real-time operation model for determining the optimal real-time release during a typhoon. The formulation of the problem is based on linear programming and the streamflow routing along a reach is modeled by using the Muskingum method of linear channel routing. Kumar et al.^23^ adopted Folded Dynamic Programming for developing optimal reservoir operation policies for flood control with channel routing based on Muskingum model imbedded within the algorithm.

Linear programming (LP) objective functions and constraints are restricted to summations of linear terms. It is the optimization technique that is most often applied in modeling reservoir/river systems as well as flood management^24^. The main advantages of linear programming are its ability to optimize large problems, its convergence towards the global optimal and the availability of efficient software packages under free license^25^). The LP is expressed as:

7 $$\begin{aligned} Minimize: z=\sum _{j=1}^{n}c_jx_j \end{aligned}$$

Subject to:

8 $$\begin{aligned} \begin{aligned}{}&\sum _{j=1}^{n}a_{ij}x_j \le b_i \ for \ i= 1,\dots ,m \\&x_j \ge 0 \ for \ j= 1,\dots ,n \\ \end{aligned} \end{aligned}$$

where *z* is the objective function, $x_j$ are the decision variables, $c_j$, $a_{ij}$, and $b_i$ are constants, *n* is the number of decision variables, and *m* is the number of constraints.

As a particular form of linear programming, network flow programming^26^, because of its intuitive formulation and short resolution time, is often used in water management applications, and is suitable for solving large-scale allocation problems of multi-reservoirs and multi-periods^3,27^. In order to applied graph theory algorithms, the hydraulic system is modeled as a directed graph where convergence and diversion points, demand locations and water sources are represented by nodes, and reservoir releases, channel flows, carryover storage and withdrawals are represented by arcs. Network flow optimization problem is expressed as:

9 $$\begin{aligned} Minimize: z=\sum _{}^{}c_{ji}q_{ij} \ for \ all \ arcs \end{aligned}$$

Subject to:

10 $$\begin{aligned} \begin{aligned}{}&\sum _{}^{}q_{ij} - \sum _{}^{}q_{ji} = 0 \ for \ all \ nodes \\&\underline{q_{ij}} \le q_{ij} \le \overline{q_{ij}} \ for \ all \ arcs \\ \end{aligned} \end{aligned}$$

where $q_{ij}$ is the flow rate in the arc connecting node *i* to node *j*; $c_{ij}$ is a cost for $q_{ij}$; $\underline{q_{ij}}$ and $\overline{q_{ij}}$ are lower and upper bound on $q_{ij}$, respectively. The only constraints allowed are the ones in the form of a “mass balance” equation.

The special structure of network flow programming inhibits the integration of the Muskingum flood routing model in the network flow problem formulation, since Eq. (6) violates the form of Eq. (10) for nodes. Considerable efforts are made to include proper modeling of hydrologic channel routing into network flow models^28^. Braga and Barbosa^29^ report on inclusion of channel routing into multiple time step optimization using an advanced Network Simplex Solver that can handle non-network side constraints required for inclusion of channel routing. Nonetheless, non-network constraints may disturb the allocation priorities as stated by Ferreira^30^ and Chou and Wu^31^. To eliminate the aforementioned disadvantages and difficulties of the use of the existing flood routing models in water resource optimization models based on a network approach, the conceptual model developed herein can be proposed. The flood routing model presented here uses the computational properties of the network flow technique, and can be coupled to a network flow structure.

## Flood routing model

### Mathematical formulation of the flood routing model

**Figure 2:**
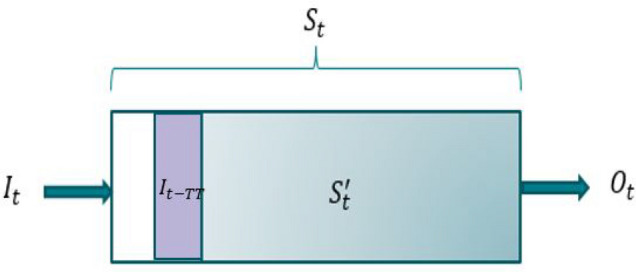
Residual storage schematic representation.

In the following, we will refer to the flood routing model developed herein as the **Residual Storage Model (RSM)**. Although inflows during immediate moments have a marginal impact on the outflow, all reservoir routing models consider that the outflow at time t is a function of the absolute reach storage (total inflow minus total outflow) at the same time. Herein, the portion of the absolute reach storage that impacts the outflow significantly is defined as the **residual storage** of a reach. Let $S_t^\prime$ denotes the residual storage at time *t*. Figure 2 illustrates the upstream and downstream of a reach, and the residual storage.

The conservation equation can be written as:

11 $$\begin{aligned} S_{t+1}^\prime =S_t^\prime +I_{t-TT}-O_t \end{aligned}$$

where *TT* is the Transit Time. This parameter represents the time from when the inflows $\{I_{t-TT}, I_{t-TT-1}, \dots , I_0\}$ impact the outflow at time t. Hence, inflows $\{I_t, I_{t-1}, \dots , I_{t-TT+1}\}$, are not used in the computation of $O_t$ and are represented by the white area in Fig. 2. The RSM considers that outflow at time t is proportional to $S_t^\prime$ and $I_{t-TT}$:

12 $$\begin{aligned} O_t=(1-\alpha )(S_t^\prime +I_{t-TT}) \end{aligned}$$

The parameters of the model are the reach’s Transit Time (*TT*), the proportionality coefficient ($\alpha \in [0:1]$ ) and the initial residual storage $S_0^\prime$.

The proportionality coefficient $\alpha$ physically represents the proportion of the residual storage that stays in the reach. It is between 0 and 1. The initial residual storage $S_0^\prime$, represents the reach storage during a steady flow.

The RSM form is a singular form of Muskingum model (see Eq. (5) with $x=0$) and is suited for long reach where the downstream flow has a limited influence on the upstream flow.

### Network flow model

In a water management problem based on a network representation, the RSM can be easily integrated if it is described as a network. Let $G=(V;E)$ be a directed single source network, with node set *V* and arc set *E*. Let *S* and *T* be the source and the sink fictive nodes of the network, respectively. The source node supplies the upstream of the system and the sink node collects the downstream flows. Convergence or diversion points and reservoirs are represented by nodes and water transfer by arcs. In fact, flows do not cross the network instantly, thus, in order to account for the dynamics of the flows, the nodes are duplicated at each time step over the duration of the simulation. In the arcs connecting those copies, the transit times and flows are implicit^32^. For an arc $e_{ij}$, *i* is the origin node, and *j* is the end node. Let $\gamma (n)$ and $\gamma ^{-1}(n)$ respectively denote the sets of the outgoing and incoming arcs of a node *n*. Each arc *e* is associated with a positive flow $\Phi _e$. For each node *n* of *V*, except for *S* and *T*, the conservation of flow is satisfied:

13 $$\begin{aligned} \forall n \in V \setminus \{ S,T \} \sum _{e \in \gamma (n)}^{}\Phi _e = \sum _{e \in \gamma (n)^{-1}}^{}\Phi _e \end{aligned}$$

Considering the reach’s transfer time *TT*, the outgoing flow from an upstream node at time *t* is connected to the reservoir node at time $t+TT$. In order to model a reach’s flood routing, an intermediate node denoted as **reservoir node** is introduced between every upstream and downstream node. The reservoir node separates the incoming flow into 2 flows: a flow corresponding the volume released from the reservoir at time *t* and a flow corresponding to the residual storage remaining in the reservoir.

**Figure 3:**
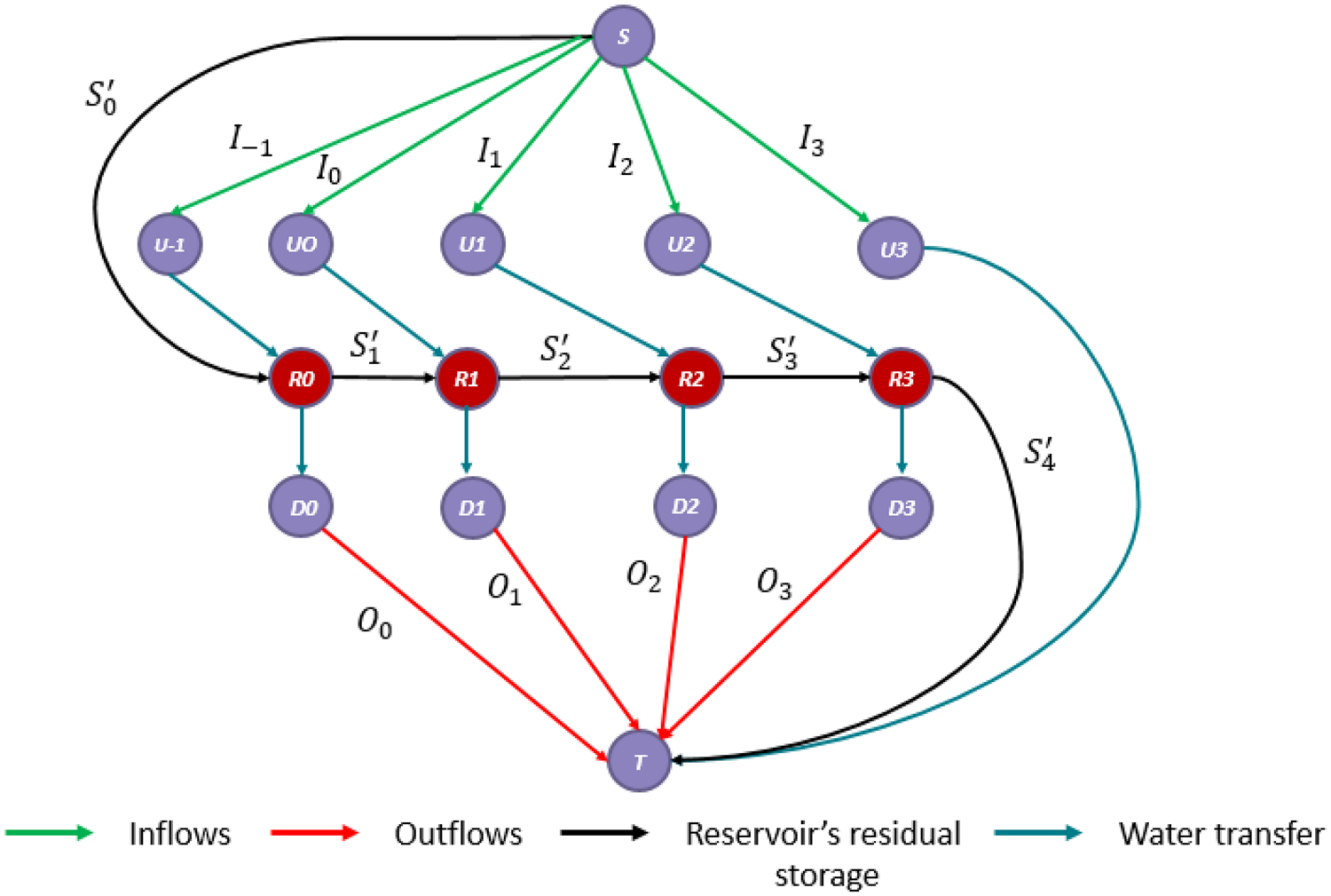
Network model corresponding to a reach, with $TT=1$ and $Horizon=4$.

Figure 3 presents an example of a network model corresponding to channel routing over one reach. To simplify the example, we considered that the transit time is equal to one-time step, and we only provided the corresponding network for a horizon of 4 times step. Nodes $U_t$ and $D_t$ represent the upstream and the downstream of the reach at time *t*, respectively. Inflow at instant *t* is represented by the flow carried by the arc $e_{SU_t }$. Inflow at instant *t* joins the reservoir at instant $t+TT$ through the arc $e_{U_tR_{t+TT}}$. The reservoir node at instant $t+TT$ preserves a proportional portion of the incoming flows: $S_{(t+TT)+1}^\prime =\alpha (S_{t+TT}^\prime +I_t)$, and releases the left portion: $O_{t+TT}=(1-\alpha )(S_{t+TT}^\prime +I_t)$.

### Calibration of the model

For the simulated outflows to be close to the measured downstream flows, the model’s parameters of the flood routing model have to be calibrated. The objective function to minimize is stated as follows:

14 $$\begin{aligned} Minimize \sum _{t=0}^{Horizon -1}(O_t^{Measured}-O_t^{RSM \ Model})^2 \end{aligned}$$

Subject to:

15 $$\begin{aligned} \begin{aligned}{}&\forall n \in V \setminus \{ S,T \} \sum _{e \in \gamma (n)}^{}\Phi _e = \sum _{e \in \gamma (n)^{-1}}^{}\Phi _e \\&S_{t+1}^\prime =\alpha (S_t^\prime +I_{t-TT}) \\&\alpha \in [0:1] \\&TT \ge 0 \\&S_0^\prime =S_{Horizon-1}^\prime \\ \end{aligned} \end{aligned}$$

where $O_t^{Measured}$ and $O_t^{RSM \ Model}$ are the measured and computed outflow at time *t*, respectively.

The unknowns of the problem are: the reach’s transit time (*TT*), the distribution coefficient ($\alpha$) and the initial residual storage $S_0^\prime$. The final residual storage $S_{Horizon-1}^\prime$ should be equal to the initial one $S_0^\prime$ in order to conserve the volume of the routed hydrograph.

In order to account for the complex objective functions involved in the calibration step, a genetic algorithm is used instead of traditional optimization algorithms. The Genetic Algorithm (GA) solver in MS Excel is coupled to a network model. Unlike classical optimization search methods, such as the simplex method and gradient-based methods, the genetic algorithm does not necessarily require well-defined functions or derivatives of functions. A genetic algorithm is a metaheuristic based on three bio-inspired operators: selection, crossover, and mutation. In the optimization model, the population in GA is the vector ($TT,\alpha ,S_0^\prime$), and the constraints are defined through the network model.

### Validation of the model

In order to evaluate the validity of the proposed model, it was tested on a hydrograph proposed by Wilson^33^, which is a standard test event that has been extensively studied by other researchers^20,34–36^. The inflows and outflows of the Wilson event, the model routed, and Muskingum outflows are given in Table 1.

**Figure 4:**
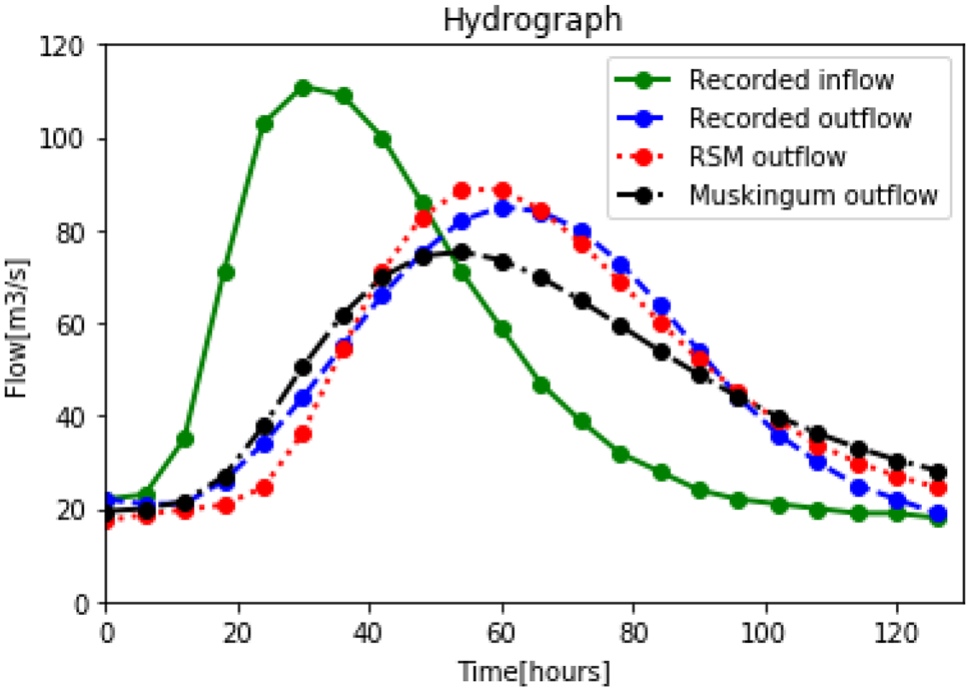
Wilson event: recorded inflow and outflow, reconstructed outflow for the RSM and the Muskingum models.

The results of the RSM and Muskingum model are illustrated in Fig. 4.

**Table 1:** Recorded and reconstructed outflow for the Wilson event.

T(*h*)	Recorded inflow ($m^3 s^{-1}$)	Recorded outflow ($m^3 s^{-1}$)	Simulated outflow (RSM) ($m^3 s^{-1}$)	Simulated outflow (Muskingum) ($m^3 s^{-1}$)
0	22	22	17.30	20.95
6	23	21	18.74	21.26
12	35	21	19.75	22.23
18	71	26	20.78	26.86
24	103	34	24.57	38.75
30	111	44	36.33	54.67
36	109	55	54.38	68.60
42	100	66	71.10	78.38
48	86	75	82.79	83.35
54	71	82	88.63	83.59
60	59	85	88.75	80.16
66	47	84	84.35	74.51
72	39	80	77.39	67.45
78	32	73	68.92	60.13
84	28	64	60.29	53.00
90	24	54	52.12	46.61
96	22	44	44.99	40.90
102	21	36	38.84	36.14
108	20	30	33.82	32.31
114	19	25	29.95	29.19
120	19	22	26.97	26.64
126	18	19	24.60	24.67

The root-mean-square (*RMS*) and the error (*Error*) are computed using Eq. (16):

16 $$\begin{aligned} \begin{aligned}{}&RMS=\sqrt{\frac{\sum _{i}^{}(Q_i^{observed}-Q_i^{model})^2}{Horizon}} \\&Error=100 \times \frac{ \sum _{i}^{} | Q_i^{observed}-Q_i^{model} | }{ \sum _{i}^{} Q_i^{observed} }\\ \end{aligned} \end{aligned}$$

For the two reconstructions of the outflow hydrograph, the root-mean-square (RMS), the error, and the model’s parameters are listed in Tables 2 and 3. The determination of *K* and *x* is performed by employing a least-square technique.

Figure 4 shows a good agreement between the RSM reconstructed outflow and the observed ones. For the Muskingum model, $C_0$ and $C_1$ are negligible compared to $C_2$ which shows that the outflow at time $t+1$ depends primarily on outflow at time *t*. Even if in the RSM model the influence of the output is only depending on the residual storage state and not directly on the output flow as in the Muskingum model, the observed results are similar. Referring to Table 2, the errors for the residual storage model and the Muskingum model is 4.73 and 4.48 respectively; and the RMS is $8.37\%$ and $9.32\%$ respectively. With the recorded outflow peak time of $t = 60 hr$, the RSM model was able to yield the same peak time with minor error. Whereas the Muskingum incorrectly predicted the peak time. The RSM also yields a very small RMS from practicing hydrologist point of view.

**Table 2:** Calibration results for the Wilson event.

	RSM	Muskingum
RMS ($m^3 s^{-1}$)	4.73	4.48
Error ($\%$)	8.37	9.32

**Table 3:** Parameter of the calibration results for the Wilson event.

	*TT*(*h*)	$\alpha$	$S_0^\prime$ ($m^3$)	$\Delta T$ (*s*)	*K*	*X*	$C_0$	$C_1$	$C_2$
RSM	11	0.94	270.13						
Muskingum	5			10800	229117.15	0.023	$12.345E-05$	0.047	0.952

**Figure 5:**
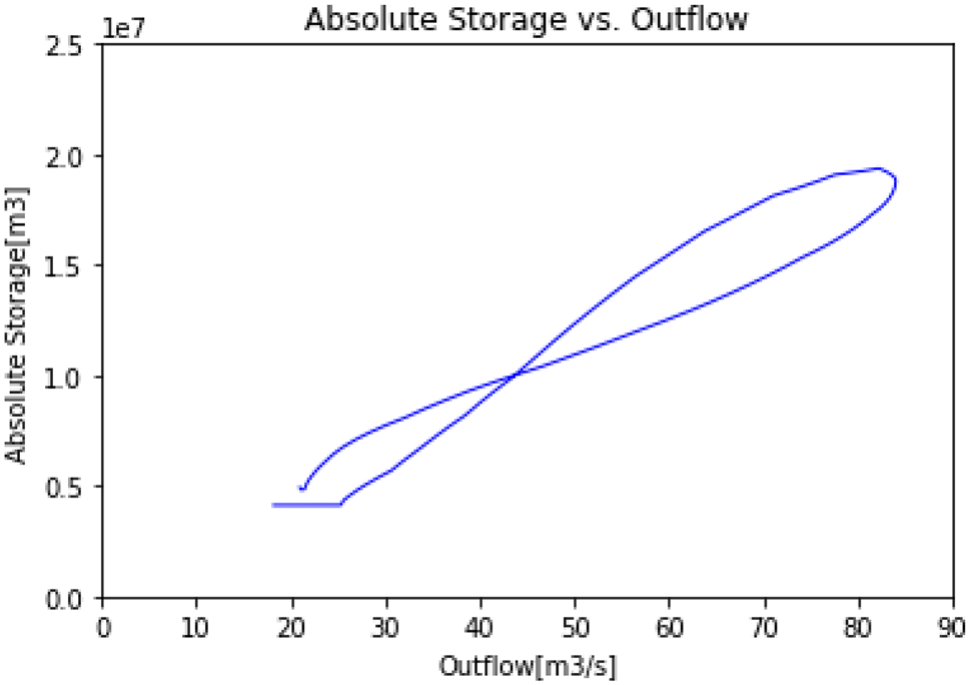
Absolute storage vs. outflow (Muskingum model).

**Figure 6:**
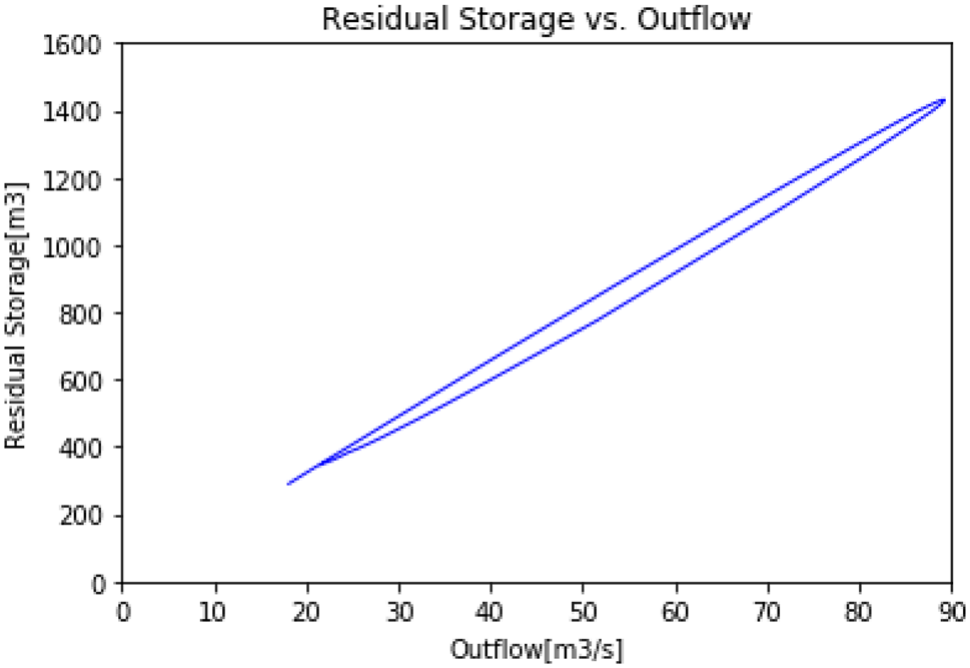
Residual storage vs. outflow (RSM).

Figures 5 and 6 represent the absolute storage of the reach plotted against the outflow for the Muskingum method, and the residual storage of the reach plotted against the outflow for the RSM, respectively. Figure 5 highlights the non-linear relationship between the absolute storage and the outflow assumed in the Muskingum model. The plot consists in a loop formed by a forward and a reverse path. The loop reflects the non-symmetrical relationship existing when the reach is storing or emptying. On the other hand, in Fig. 6, the loop is nearly closed and a highly linear relationship between the outflow and the residual storage can be observed. Figures 5 and 6 emphasize that the outflow is more linearly correlated to the residual storage than the absolute storage of a reach.

## Application

### Application on the Arrats river

**Figure 7:**
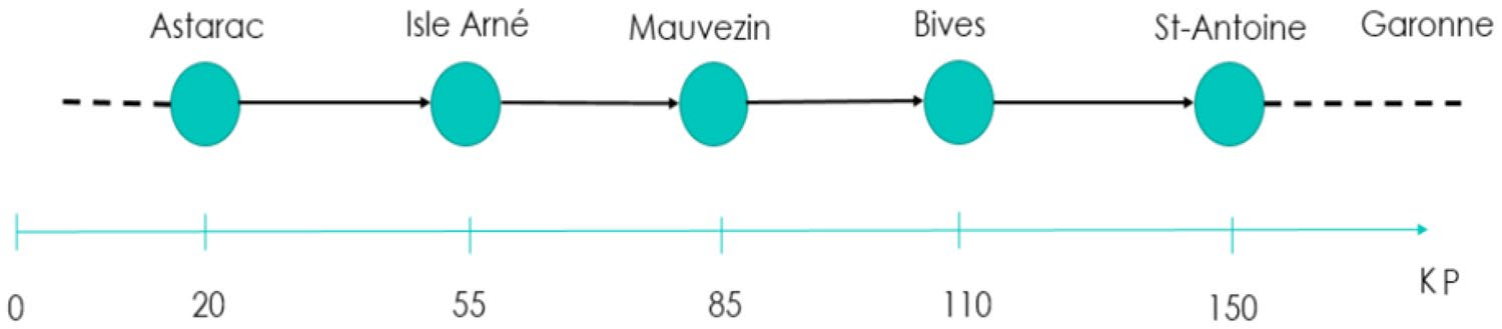
Synoptic of the Arrats’ river (with KP indicating the kilometric point).

**Figure 8:**
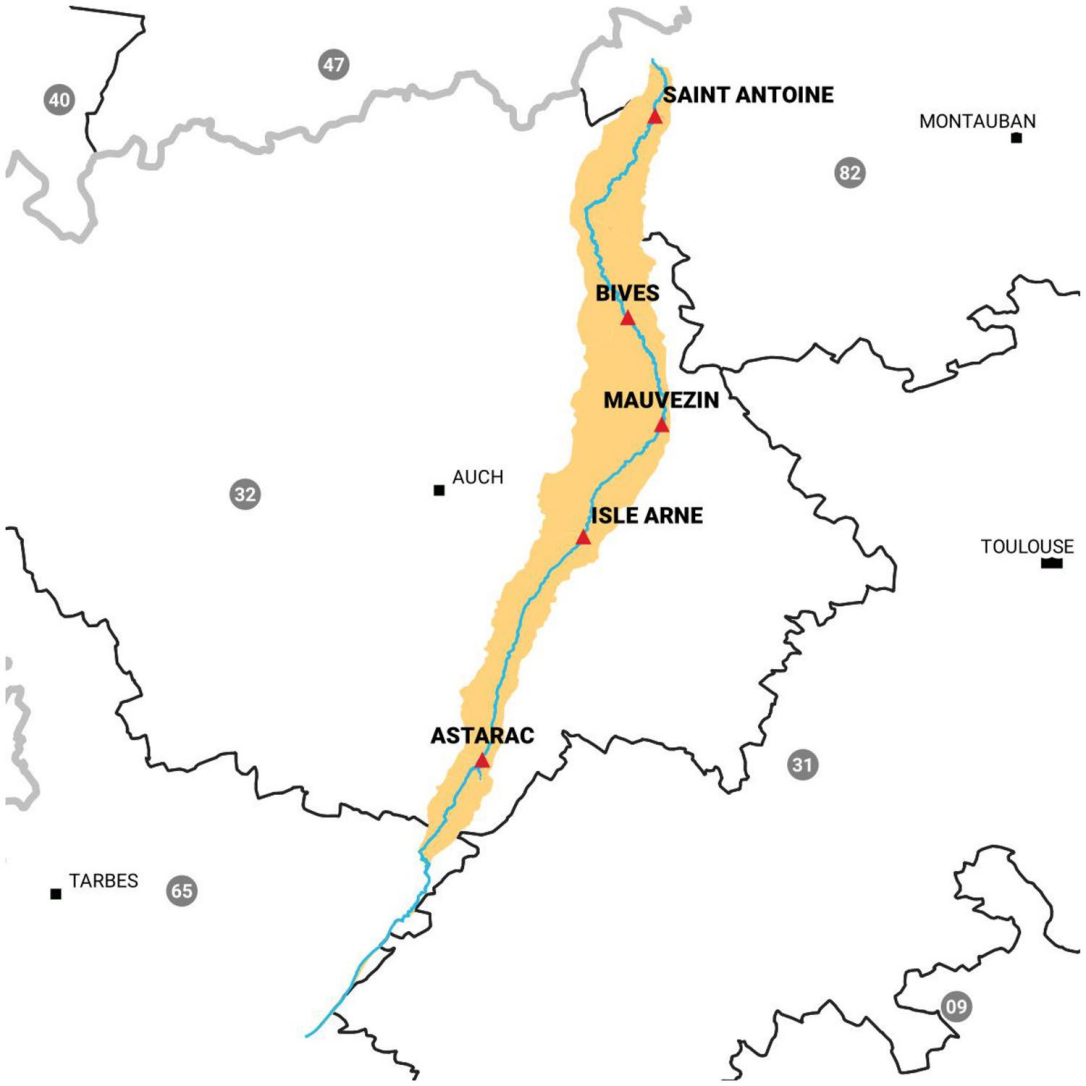
Map of the Arrats’ river and the hydrometric stations created with QGis version 3.16 (www.qgis.org).

The RSM developed herein has been applied on a reach of the Arrats river. The Arrats river is an affluent of the Garonne river in the south west of France. The river is equipped with five hydrometric stations: Astarac (S1), Isle Arne (S2), Mauvezin (S3), Bives (S4), and St-Antoine (S5). Figure 7 presents a synoptic of the river and the distances between the hydrometric stations, and Fig. 8 presents a map of the river and the hydrometric stations. In this case of study, we will focus on the last reach between Bives and St-Antoine, with a length of 40*km*. The aim of this study is to compare the outflow routed by the RSM and the measured outflow.

#### Calibration

The hydrograph chosen for the calibration is a 200 hours’ event measured in the Bives and St-Antoine hydrometric stations during the period $03/07/2018 - 11/07/2018$. The total simulation time was 200 hours starting at $t=0h$, using a computation interval of one hour. The RSM was calibrated and the parameters $TT, \alpha , and S_0^\prime$ were found. The calculated parameters were $TT=11h, \alpha =0.82, and S_0^\prime =6.23m^3$. In order to compare the RSM and the Muskingum model, the latter was also calibrated, and the calculated parameters were $K=58412.7, x=0.07$ and $\Delta T=10580 s\approx 3h$.

**Figure 9:**
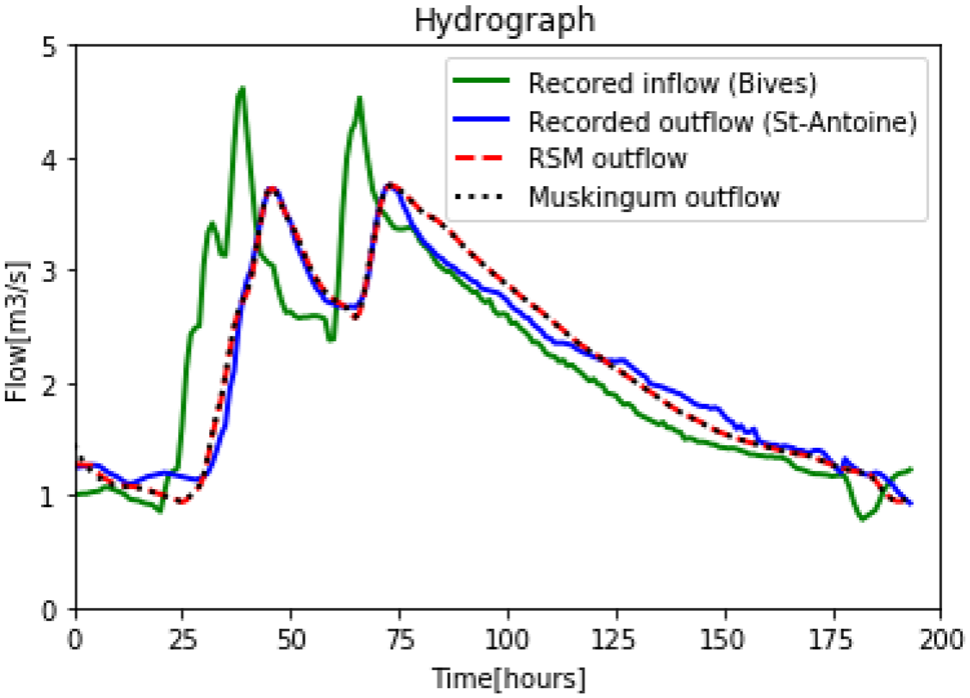
Calibration: Recorded inflow in Bives, recorded outflow in St-Antoine, reconstructed outflow for the RSM and the Muskingum models.

Figure 9 shows that the calibration of the RSM and Muskingum model indicates good performance. In fact, the errors for RSM and Muskingum model are only of: $5.07 \%$ and $4.48 \%$, respectively.

#### Simulation

**Figure 10:**
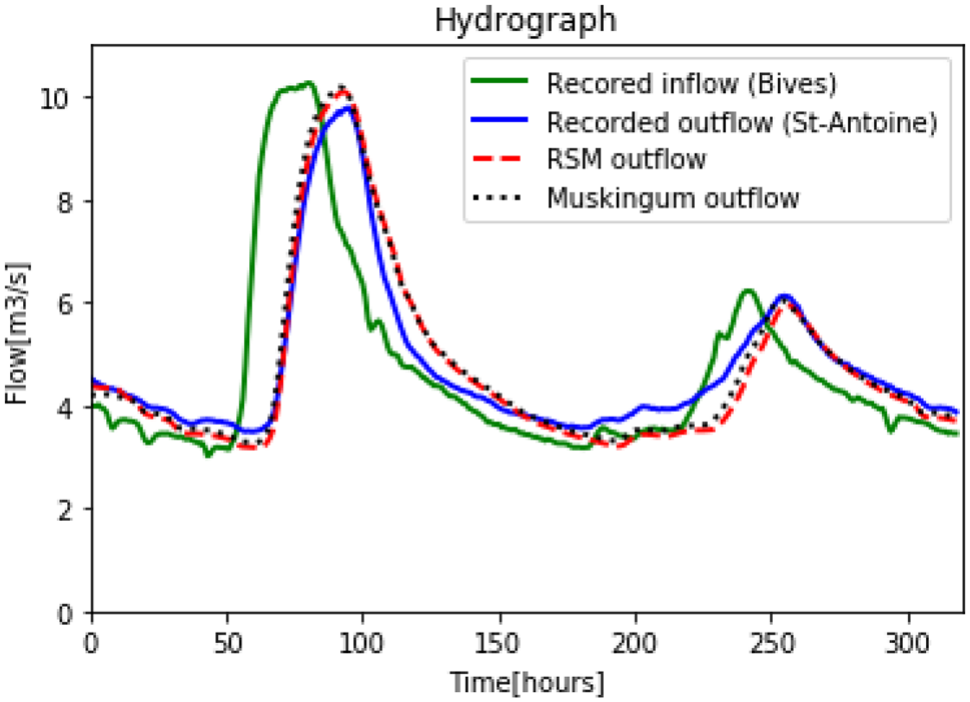
Simulation: Recorded inflow in Bives, recorded outflow in St-Antoine, reconstructed outflow for the RSM and the Muskingum models.

Once the RSM and Muskingum model were calibrated on the last reach of the Arras river, they were tested on other hydrological events in order to examine the constancy of the methodology and the parameters found in the calibration stage. The hydrograph chosen for the simulation was a 320 hours’ event measured in the Bives and St-Antoine hydrometric stations during the period $06/05/2018 - 20/05/2018$. The parameters used to simulate the outflow at St-Antoine, were the ones found in the calibration stage. Recorded inflow, recorded outflow and simulated outflows with RSM and Muskingum model are illustrated in Fig. 10.

Figure 10 shows that the outflow simulated with the RSM is close to the measured one. It also shows that RSM results are similar to Muskingum’s results. The errors and root-mean-square for RSM and Muskingum model are: $(7.1 \% ; 0.44 m^3 s^{-1})$ and $(6.6 \% ; 0.43 m^3 s^{-1})$, respectively, see Table 4 and Table 5. The simulation results analysis shows that the proposed routing model can be readily calibrated and provides pertinent results. The Muskingum routing model is convenient in deriving the outflow with given inflow hydrograph, however the result can be greatly affected by inappropriate K and X. There is no general rule of thumb of how the Muskingum K and X should be determined, thus uncertainties remain if these parameters are incorrected used. The routing method incorporated with network flow presented in this paper is to minimize deviations between the observed and derived hydrograph before applying for outflow hydrograph derivation.

**Table 4:** Simulation results for the Arrat’s reach.

	RSM	Muskingum
RMS ($m^3 s^{-1}$)	0.44	0.43
Error ($\%$)	7.1	6.6

**Table 5:** Parameter of the simulation results for the Arrat’s reach.

	*TT*(*h*)	$\alpha$	$S_0^\prime$ ($m^3$)	$\Delta T$ (*s*)	*K*	*X*	$C_0$	$C_1$	$C_2$
RSM	11	0.82	16						
Muskingum	5			10580	58412.7	0.0717	0.0185	0.1593	0.8222

### Operative results

**Figure 11:**
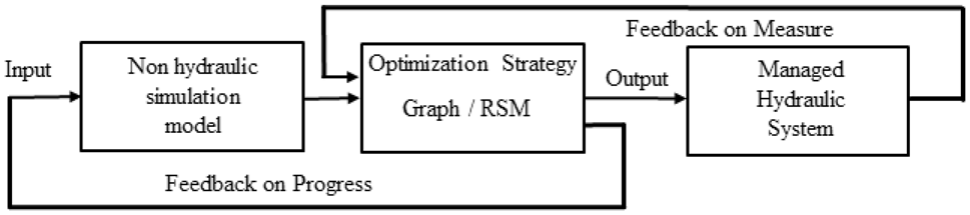
Scheme for the optimization strategy integrating hydraulic models.

The RSM model has been integrated into the operational management tool Rio used by the -CACG following the functional principle described in the Fig. 11. Feedback on measurement are operated directly by the optimization model. The feedback on progress loop previously presented on the Fig. 1, is reduced to non-hydraulic models such as streamflow or withdrawal models. This principle is more detailed and illustrated in Tahiri et al.^37^ where it was implemented in order to improve initial conditions for streamflow prediction using rainfall reconstruction algorithms.

**Figure 12:**
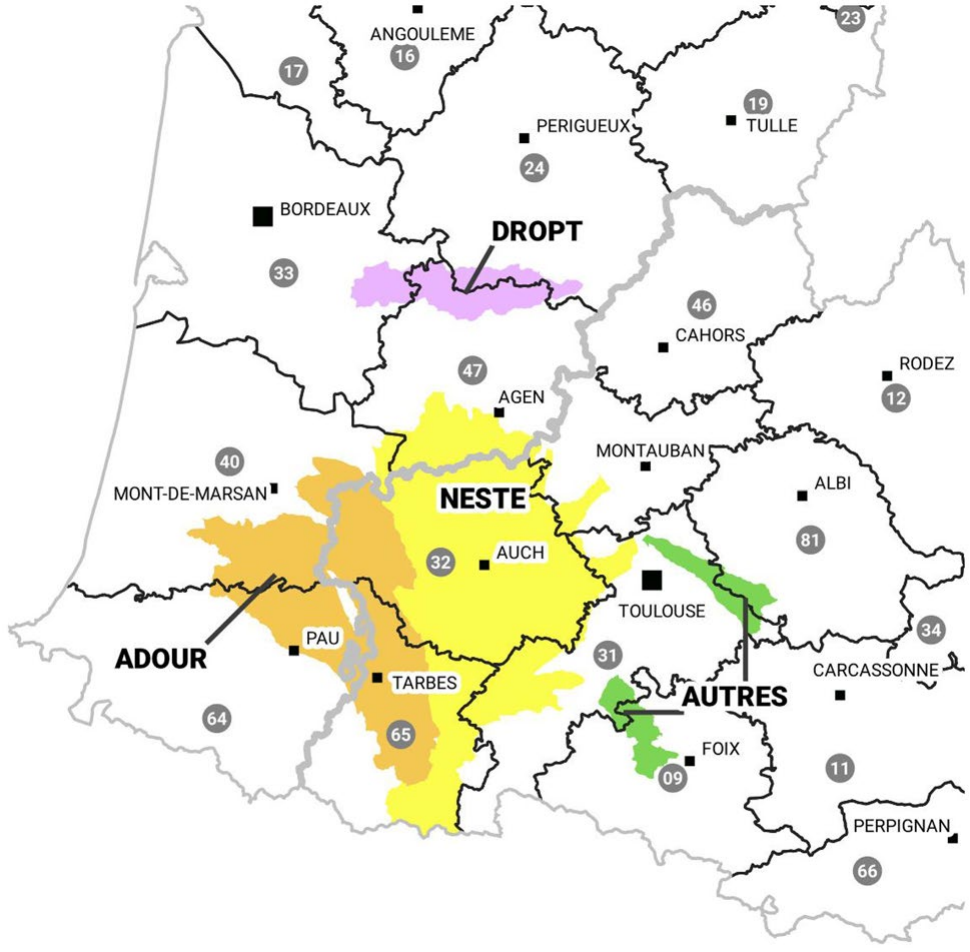
Hydrographic perimeter of the operational water resource system, created with QGis version 3.16 (www.qgis.org).

RSM model is actually in operation on about thirty managed rivers in the south of France presented in Fig. 12. The Neste system is composed by the rivers in the yellow zone which form a singular system alimented by a single canal. All these rivers are left tributaries of the Garonne river except the most westerly river that provides another river named Adour (tributaries represented in orange in Fig. 12). The green and purple rivers in Fig. 12 constitute part of the right tributaries of the Garonne river.

Rio integrates models to managed dams and canal intakes:

- 194 watersheds are modeled by rainfall-runoff model based on 3 interconnected reservoirs.
- 84 RSM models are used to represent the flow dynamics.
- Withdrawals are partially measured and forecasts are statistically interpolated.

Mid-term weather forecasts are obtained from GFS model (10 days). For the current day French model AROME rainfall forecasts are used. For both, 8 representative weather stations are consulted.

**Figure 13:**
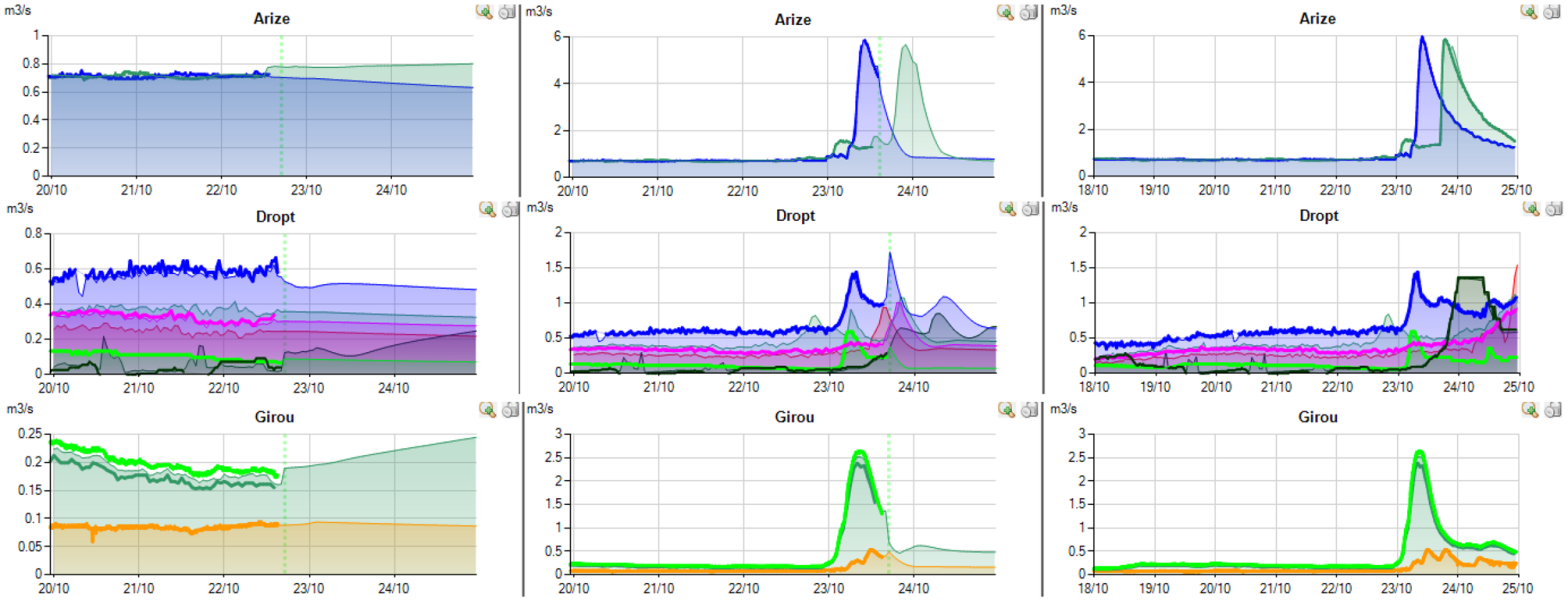
Right tributaries of the Garonne dashboards screenshots.

**Figure 14:**
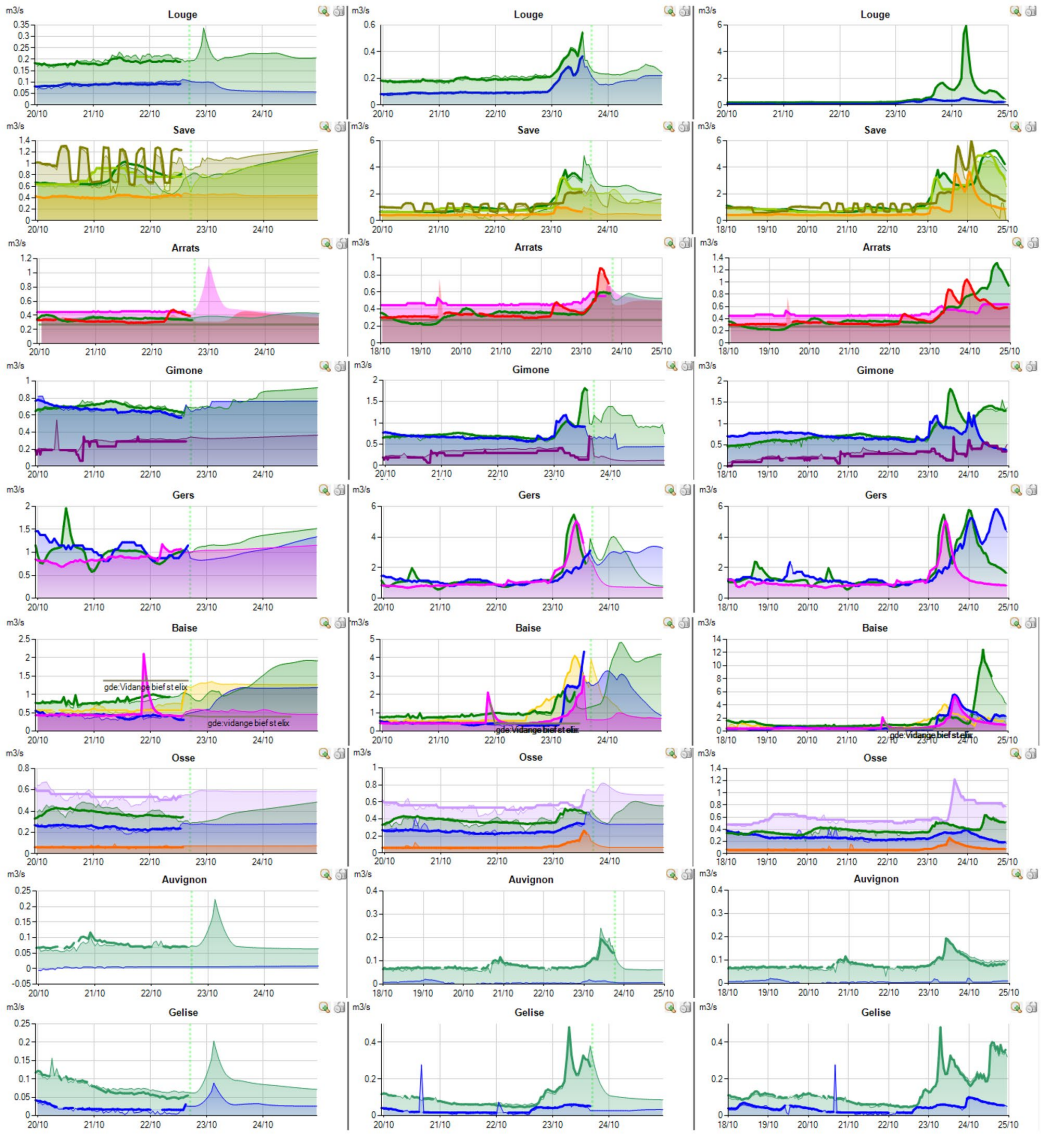
Left tributaries of the Garonne dashboards screenshots.

**Figure 15:**
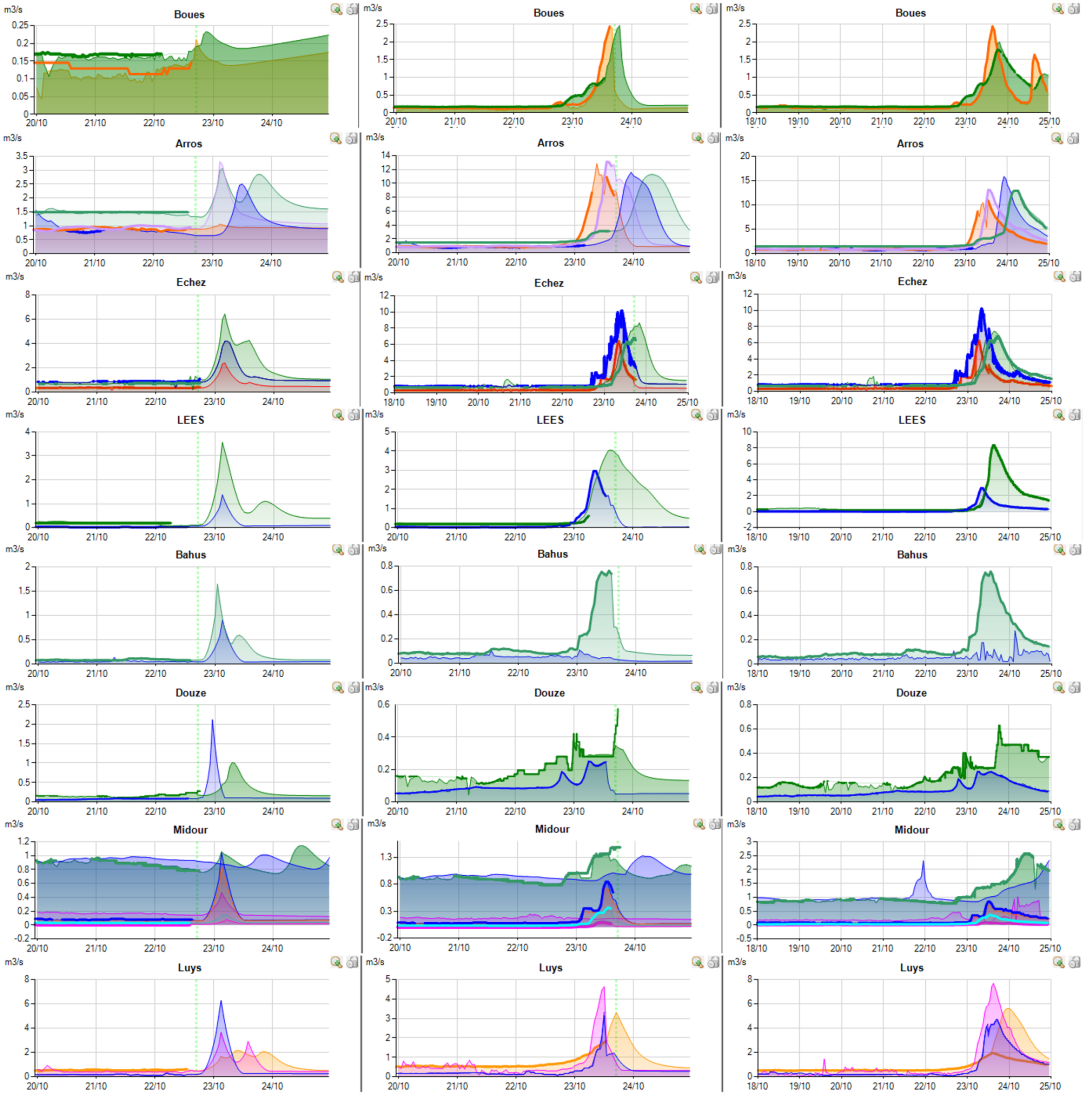
Adour tributaries dashboards screenshots.

The rainfall event presented herein (Figs. 13, 14, 15) for 20 of the managed rivers occurred between the 22/10/2019 and the 25/10/2019. During this period, weather forecast was particularly uncertain due to the stormy conditions. On the screenshot herein, discharge measurements are colored, full and bold lines. The model outputs are represented by a surface of the same color as that used for the measurement station. Vertical dotted green line corresponds to the time of the screen capture. In order to be accurate, the model outputs have to match the measured flows before this line and the forecast flows after. For each river, three screenshots from 22/10/2019 at 6PM, 3/10/2019 at 6PM and 25/10/2019 at 10AM are displayed.

## Conclusion

Real-time operation of multireservoir systems requires flood routing in river reaches. This issue has usually been solved by coupling a simulation model to the optimization model when it is based on an evolutionary approach. Conceptual models for channel routing like the Muskingum model can also be imbedded within the mathematical formulation of the optimization algorithm when it is linear. Nonetheless, even if network flow programming is a special form of linear programming, its special structure inhibits the integration of the Muskingum flood routing model in the network flow problem formulation. A new flood routing model destined primarily to be coupled with a network flow structure is developed. The routing model is considered as a network flow and the parameters are calibrated using a genetic algorithm. The model was approved on the Wilson standard test, studied by other researchers and known to present a nonlinear relationship between weighted discharge and storage. The methodology was tested and provided satisfying results on reaches of many rivers in the south west of France. This flood routing model was integrated to various operational systems since two years on several rivers and more recently experimented on the whole system for which calibration and weather forecast accuracy improvements are needed and are actually under studies. Thus, sensibility analysis hasn’t been studied in this paper, because of its importance, it is considered as the main perspective of this work.
